# Judgements about double-embedded relative clauses differ between languages

**DOI:** 10.1007/s00426-018-1014-7

**Published:** 2018-04-23

**Authors:** Stefan L. Frank, Patty Ernst

**Affiliations:** Centre for Language Studies, Radboud University Nijmegen, P.O. Box 9103, Nijmegen, 6500 HD The Netherlands

## Abstract

When the middle verb phrase is removed from an English double-embedded sentence, the remainder of the sentence is read faster in spite of the ungrammaticality. It has been shown that this “missing-VP effect” is reversed in German and Dutch. The current study demonstrates that the same cross-linguistic difference holds for sentences judgments: Native speakers consider English double-embedded sentences more comprehensible and acceptable when the middle verb phrase is removed, whereas the same is not the case in Dutch. This interaction between language and grammaticality also appears in a within-subjects replication that tests Dutch native speakers in both languages. These results, in combination with earlier findings, give rise to a hybrid account according to which the missing-VP effect is caused by properties of the language as well as properties of working memory.

**Electronic supplementary material:**

The online version of this article (10.1007/s00426-018-1014-7) contains supplementary material, which is available to authorized users.

## Introduction

A grammaticality illusion occurs when an ungrammatical sentence is perceived as acceptable (Phillips, Wagers, & Lau, 2011). One well-known example of such an illusion is the so-called missing-VP effect: When a double center-embedded relative clause structure, as in (1a), is turned into an ungrammatical string by removing the middle verb phrase (VP), as in (1b), its subjective acceptability does not appear to be negatively affected. Indeed, Gibson and Thomas (1999) found that the grammatical and ungrammatical sentence versions are equally difficult to understand; and other studies even report higher comprehensibility or acceptability ratings for double-embedded structures from which the second VP is missing (Christiansen & MacDonald, 2009; Gimenes, Rigalleau, & Gaonac’h, 2009). Gibson and Thomas (1999) ascribe this missing-VP effect to working-memory limitations that cause the prediction of the second VP to be structurally forgotten when three consecutive noun phrases are processed.

- The exciting book that the popular author who the reviewers meticulously criticized very confidently published was missing a number of pages.
- The exciting book that the popular author who the reviewers meticulously criticized was missing a number of pages.

The missing-VP effect has also been observed in word-reading times: Vasishth, Suckow, Lewis, and Kern (2010) found that the final verb and post-verbal region are read faster in the ungrammatical condition than in correct double-embedded sentences, at least in English. Interestingly, this effect is reversed when German native speakers read German double-embedded sentences. In this case, the grammar violation caused a slowdown in reading. Vasishth et al. (2010) argue that this is because German has verb-final relative clauses, which is to say that the verb is always located at the end of a relative clause. Consequently, speakers of German often encounter sentences where the verb appears late, which may increase their ability to keep verb predictions in working memory so that they are less prone to structural forgetting than English speakers.

Frank, Trompenaars, and Vasishth (2016) replicated Vasishth et al.’s (2010) reading-time results in Dutch; a language that, like German, has verb-final relative clauses. Again, there was no missing-VP effect in the verb-final language. However, the effect reappeared when Dutch or German native speakers were tested in English (as a second language), suggesting that the cross-linguistic difference is not due to properties of the speakers (i.e., higher verbal working-memory capacity for Dutch and German speakers compared to English speakers) but is caused by properties of the languages. For example, Dutch and German word order makes consecutive VPs much more common in those languages than in English. Sensitivity to these statistics could speed up the processing of three consecutive VPs in Dutch/German compared to English.

These results do not imply that a missing-VP effect can never arise in verb-final languages. As a case in point, Häussler and Bader (2015) found longer reading times in grammatical double-embedded German sentences compared to versions without the second VP, if the entire structure was presented as a complement clause (as in: “I believe that the exciting book that ...”). The present study investigates sentences without such a complementizer, that is, sentences like (1a) and (1b), for which the absence of the missing-VP effect in Dutch and German is well established, at least when reading time is the dependent variable.

Neither Vasishth et al. (2010) nor Frank et al. (2016) asked participants to rate the sentence stimuli so the question remains whether the cross-linguistic difference between English and German/Dutch also appears in subjective judgments. Häussler and Bader (2015) claim that categorical grammaticality judgments in German reveal a missing-VP effect, based on the fact that “in a substantial number of cases” (p. 10) the missing-VP sentences are judged to be grammatical. However, the acceptance rate was much higher for grammatical sentences (81 versus 33%, for sentence without an initial complementizer) which stands in stark contrast to previous studies in English and French that found comprehensibility or acceptability ratings to be equal or lower in the grammatical condition (Christiansen & MacDonald, 2009; Gibson & Thomas, 1999; Gimenes et al., 2009). Therefore, it seems premature to conclude that there is a missing-VP effect in German grammaticality judgments.

The current study investigates whether the missing-VP effect occurs in judgments about Dutch equivalents to sentences like (1a) and (1b). If the effect depends on the language’s statistical properties, as Frank et al. (2016) argue, the cross-linguistic difference may be restricted to reading times, while sentence comprehensibility or acceptability remain relatively unaffected. This is because word-reading times are automatically tuned to the probabilistic (i.e., statistical) information conveyed by each word (e.g., Smith & Levy, 2013). Although a recent study by Lau, Clark, and Lappin (2017) has shown that sentence acceptability ratings, too, correlate with the probabilities that follow from the statistics of a language’s word-order patterns, ratings are likely to be less probability sensitive than reading times because they result from conscious, deliberative processes.

In addition, we investigate if the difference between verb-initial and verb-final languages also appears when sentence comprehension is facilitated by semantic support. The sentence items of Vasishth et al. (2010) and Frank et al. (2016) contained nouns and verbs that allow for any combination of agent, action, and patient. For example, the sentence “The mother who the daughter who the sister found frightened greeted the grandmother” can only be understood through syntactic analysis because the meaning of the individual words do not provide any cue about who does what to whom. In sentence (1a), on the other hand, it stands to reason that it is the book that was missing a page and that the reviewers did the criticizing, even if word order is ignored. Christiansen and MacDonald (2009) demonstrated that the missing-VP effect occurs in English irrespective of whether such semantic support is present, but in German and Dutch it has only been investigated on semantically neutral sentences. Possibly, semantic support leads to more shallow parsing (Sanford & Sturt, 2002) or to prioritizing semantic over syntactic analysis (Townsend & Bever, 2001). This, in turn, could mean that the language’s word order (a purely syntactic parameter) is no longer relevant to the presence of the missing-VP effect.

We had participants rate sentences on their comprehensibility (as did Gibson & Thomas, 1999, and Gimenes et al., 2009) as well as their acceptability (Christiansen & MacDonald, 2009; Häussler & Bader, 2015). It is conceivable that effects diverge between these two dependent variables, for example because the reading slowdown on Dutch ungrammatical sentences causes a decreased sense of acceptability without affecting perceived comprehensibility. However, our expectation was that comprehensibility and acceptability show similar patterns because they form merely alternative measures of participants’ underlying sentence-reading experience. If effects on the two measures indeed show similar patterns, this can, therefore, be considered converging evidence for the effect of sentence grammaticality.

In Experiment 1, native Dutch-speaking participants rated Dutch double-embedded sentences, similar to the English items from the Gibson and Thomas (1999) study. Results showed that there was no missing-VP effect: grammatically correct sentences were rated as more comprehensible and more acceptable than the ungrammatical versions. Experiment 2 is identical to Experiment 1 except that it has English stimuli and participants are native speakers of English. Consistent with Gibson and Thomas (1999), sentences with a missing second verb phrase were rated as more acceptable and comprehensible than grammatically correct sentences. Finally, Experiment 3 replicates Experiments 1 and 2 in a within-subjects design by presenting both Dutch and English items to native Dutch speakers of English as a second language. This experiment confirmed the findings from the first two experiments: The presence of the missing-VP effect is language dependent.

## Experiment 1: Dutch

### Method

#### Materials

Twelve target sentences were constructed, inspired by the English double center-embedded object relative (OR) structures from Gibson and Thomas (1999). The main difficulty with creating Dutch equivalents is that Dutch relative clauses are ambiguous between subject and object relative. This ambiguity is resolved at the verb if the main-clause and subordinate-clause nouns differ in number: The subject must be the noun that agrees in number with the verb. However, the verb appears at the end of the relative clause and up to that point there is a strong preference for a subject-relative (SR) reading in absence of other (e.g., semantic) cues (Mak, Vonk, & Schriefers, 2002, 2006). For this reason, all our sentences had an inanimate first noun phrase (NP), facilitating an OR reading. Further, only the third NP and its verb are plural, which syntactically disambiguates towards OR.^1^ In the “General discussion”, we will return to the issue of Dutch SR/OR-ambiguity and how it plays out in our stimuli.

The general structure of grammatical target sentences is depicted in (2) below. Note that the numbering of NPs and verb phrases (VPs) denotes their linear order rather than subject–verb dependencies. The form of the relative pronoun “die” or “dat” depends on the grammatical gender (common or neuter, respectively) of the modified noun.

(2) NP1$_\text {sing,inanim}$*die/dat* NP2$_\text {sing}$*die/dat* NP3$_\text {plu}$ VP1$_\text {plu}$ VP2$_\text {sing}$ VP3$_\text {sing}$.

Each target sentence comes in four sentence structure conditions: the complete and grammatically correct sentence (condition V0) and without the first, second, or third VP (conditions V1, V2, and V3, respectively). Table 1 shows one example in all four conditions. All twelve target sentences are listed in Appendix A.

**Table 1:** Example item from Experiment 1 in each condition, with English gloss for the grammatical (V0) condition.

Condition	Sentence
V0	Het spannende boek dat de populaire schrijver die de recensenten
	nauwlettend bekritiseerden met veel vertrouwen publiceerde miste een aantal pagina’s.
	*The exciting book that the popular author who the reviewers*
	*meticulously criticized with much confidence published missed a number_of pages.*
	“The exciting book that the popular author who the reviewers
	meticulously criticized very confidently published was missing a number of pages.”
V1	Het spannende boek dat de populaire schrijver die de recensenten
	met veel vertrouwen publiceerde miste een aantal pagina’s.
V2	Het spannende boek dat de populaire schrijver die de recensenten
	nauwlettend bekritiseerden miste een aantal pagina’s.
V3	Het spannende boek dat de populaire schrijver die de recensenten
	nauwlettend bekritiseerden publiceerde met veel vertrouwen.

In condition V0, NP1 is the unambiguous subject of VP3. The innermost relative clause “NP2$_\text {sing}$ die/dat NP3$_\text {plu}$ VP1$_\text {plu}$” is unambiguously OR because the plural verb must agree with the plural subject NP3. The outermost relative clause “NP1$_\text {sing}$ die/dat NP2$_\text {sing}$ VP2$_\text {sing}$” is syntactically ambiguous between SR and OR but the choice of nouns and verbs was such that only an OR reading made sense. Semantic constraints also prevent (most) other combinations of subject, nouns and verbs. Consequently, when one VP is removed, it is semantically evident which of the three NPs is left without a verb.

In condition V3, the final verb phrase (VP2) can be interpreted as an intransitive because its verb is always optionally transitive. As can be seen in Table 1, this condition involves a word-order change within VP2. This is necessary because word-order constraints in Dutch would make condition V3 highly unacceptable if the original order of VP2 were retained.

We constructed 48 filler sentences in addition to the targets. Twelve of these were intentionally ungrammatical so that the ungrammaticalities are not restricted to target sentences. Gibson and Thomas (1999) did not include ungrammatical fillers, but Christiansen and MacDonald (2009) did.

Four lists were constructed, each containing 12 targets and 48 fillers. Each target item occurs in all four conditions across the lists but only once in each list, and the conditions occur equally often in a list. No two targets directly followed one another in a list, and the first and last two sentences of each list were fillers. To counter potential order effects, four more lists were created by reversing the order of the original lists, making a total of eight lists. Each participant was randomly assigned to one of the lists.

#### Procedure

The experiment was conducted online as a Qualtrics questionnaire. Participants were first asked to confirm that their native language is Dutch. In case of a negative response, the questionnaire would immediately halt. Otherwise, participants provided basic demographic information and listed any reading difficulties they may have. After a brief explanation of the task, participants were presented with the 60 target and filler sentences but only one sentence was visible at a time. Each sentence had to be rated on two 7-point scales. The first scale was labeled “Zeer onbegrijpelijk/Zeer begrijpelijk” (“Very incomprehensible/Very comprehensible”) and the second was labeled “Zeer onacceptabel/Zeer acceptabel” (“Very unacceptable/Very acceptable”). The participants could not return to a previous item or skip a sentence but it was possible to close the questionnaire before completion, in which case the incomplete data were recorded. Participants who rated all 60 sentences were then asked to give their impression about the experiment’s goal.

#### Participants

Adult, native speakers of Dutch were recruited via social media. They did not receive any reward for their participation. 54 people initiated the experiment but we only analyzed data from participants who rated at least two items in each of the four conditions (i.e., at least 8 of the 12 target items). One participant’s data was discarded because the response to the experiment goal question indicated awareness of the missing-VP effect. Data were also discarded from one participant who reported being dyslectic. This left 45 participants (36 females, age range 19–62 years, mean age 33.2). This should be sufficient to detect an effect of the same size as in Gibson and Thomas’s (1999) study, who tested 40 participants in a comprehensibility rating task using materials very similar to ours, with the same conditions and the same number of target sentences.

### Results

Table 2 shows the mean comprehensibility and acceptability ratings for each condition. At first glance, there does not appear to be any grammaticality illusion: The scores in grammatical condition V0 are higher than in the three ungrammatical conditions. This was confirmed by an ordinal mixed-effects regression analysis, using the R (R Core Team, 2015) package ordinal (Christensen, 2015), which included by-subject and by-item random intercepts and random slopes of Condition. As is clear from the fitted regression model in Table 3, all three ungrammatical conditions result in significantly lower comprehensibility and acceptability scores than condition V0. Qualitatively identical results were obtained when only the 42 participants who rated all 12 target items were included in the analysis (see Appendix B).

**Table 2:** Mean and by-subject standard errors of ratings in Experiment 1, on a scale from 1 (very incomprehensible/unacceptable) to 7 (very comprehensible/acceptable)

Rated aspect	Condition			
	V0	V1	V2	V3
	M (SE)	M (SE)	M (SE)	M (SE)
Comprehensibility	4.04 (0.18)	3.56 (0.19)	3.39 (0.18)	2.98 (0.17)
Acceptability	2.79 (0.18)	2.41 (0.18)	2.32 (0.15)	2.04 (0.15)

**Table 3:** Fixed-effect coefficients *b* (with condition V0 as reference level) and their *z*-scores and *p* values from regression analysis of ratings in Experiment 1.

Condition	Rated aspect					
	Comprehensibility			Acceptability		
	*b*	*z*	*p*	*b*	*z*	*p*
V1	− 0.85	− 3.16	0.002	− 0.84	− 2.31	0.021
V2	− 1.04	− 3.24	0.001	− 0.85	− 2.24	0.025
V3	− 1.56	− 5.35	< 0.0001	− 1.52	− 4.64	< 0.0001

### Discussion

Frank et al. (2016) found that the missing-VP effect does not appear in reading times on Dutch sentences. The current results are consistent with this finding, and extend it to ratings of sentence comprehensibility and acceptability. They contrast sharply with Gibson and Thomas’s (1999) results in an English sentence comprehensibility rating task, where scores in conditions V0 and V2 did not significantly differ but were higher than those in conditions V1 and V3. Likewise, Christiansen and MacDonald (2009) report that acceptability was higher in the ungrammatical condition V2 than in V0, and Gimenes et al. (2009) found the same for comprehensibility ratings in French.

It stands to reason that the difference between our results and those from earlier studies in English and French is caused by the difference in language of presentation. However, there are a number of confounds that prevent such a conclusion. In particular, our study was conducted online, while the experiments by Gibson and Thomas (1999), Christiansen and MacDonald (2009), and Gimenes et al. (2009) took place under more controlled conditions, in the presence of the experimenter. To investigate if the English missing-VP effect also occurs under the precise conditions of Experiment 1 and with items that are as similar as possible, Experiment 2 repeats Experiment 1 but with English translations of the Dutch items and native English-speaking participants.

## Experiment 2: English

### Method

#### Materials

The sentence items were (approximate) translations into English of the Dutch stimuli from Experiment 1 (see Appendix A for the list of target sentences). The fillers were translated too, which required introducing different grammatical errors if the Dutch errors could not be translated. The item presentation order across eight lists was identical to that of Experiment 1.

#### Procedure

The experimental procedure was identical to that of Experiment 1 except that the entire questionnaire was presented in English and participants had to confirm that English was their native language.

#### Participants

Adult native English speakers were recruited via social media and other internet fora. They did not receive any reward for their participation. After 100 people participated, we discovered that four of the eight lists were not presented correctly. The corresponding data were discarded and 41 additional participants were recruited, evening out the lists. From the two recruitment sessions combined, we only kept data from participants who completed at least two items in all four conditions. Data from two participants who reported being dyslectic were discarded, leaving a total of 38 participants (23 females, age range 18–69 years, mean age 35.3), which is very close to the number of participants (40) tested by Gibson and Thomas (1999) so should suffice to detect effects of similar size. None of the participants indicated any awareness of the missing-VP effect or grammaticality illusions.

### Results

The mean comprehensibility and acceptability scores, presented in Table 4, suggest that participants experienced a grammaticality illusion when the second verb phrase was missing: The scores in condition V2 are slightly higher than in V0, whereas conditions V1 and V3 do not result in higher ratings except for V1 acceptability. This missing-VP effect was indeed confirmed by an ordinal mixed-effects regression analysis, as can be seen in Table 5: Only in the V2 condition are comprehensibility and acceptability ratings significantly higher than in the V0 reference condition. If only the 33 participants who rated all 12 target items are included in the analysis, results are qualitatively similar although the difference in V0 and V2 acceptability between is now only marginally significant (see Appendix B).

**Table 4:** Mean and by-subject standard error of ratings in Experiment 2, on a scale from 1 (very incomprehensible/unacceptable) to 7 (very comprehensible/acceptable)

Rated aspect	Condition			
	V0	V1	V2	V3
	M (SE)	M (SE)	M (SE)	M (SE)
Comprehensibility	3.45 (0.18)	3.37 (0.16)	3.99 (0.18)	3.16 (0.20)
Acceptability	2.52 (0.18)	2.67 (0.16)	2.86 (0.16)	2.36 (0.17)

**Table 5:** Fixed-effect coefficients *b* (with condition V0 as reference level) and their *z* scores and *p* values from regression analysis of ratings in Experiment 2

Condition	Rated aspect					
	Comprehensibility			Acceptability		
	*b*	*z*	*p*	*b*	*z*	*p*
V1	− 0.14	− 0.55	> 0.5	0.42	1.25	> 0.2
V2	0.69	2.46	0.014	0.66	1.98	0.048
V3	− 0.44	−1.66	0.097	− 0.35	− 1.05	> 0.2

### Discussion

Experiment 2 formed a conceptual replication of the paper-and-pencil study by Gibson and Thomas (1999). They found evidence for a missing-VP effect, in that removing the second VP from double-embedded sentences did not significantly affect comprehensibility, whereas removing either the first or third VP resulted in significantly reduced ratings. Our results are somewhat different, but consistent, with this pattern. Instead of lower scores in the V1 and V3 conditions (compared to V0), we found higher comprehensibility and acceptability ratings in condition V2 compared to V0. Although one may be tempted to take this as evidence for a grammaticality illusion in all three ungrammatical conditions, the fact that most mean scores in the V1 and V3 conditions are numerically lower than for grammatical sentences (V0) is suggestive of a weak effect of the syntactic violation. Importantly, ratings are significantly higher in condition V2 which confirms Gibson and Thomas’s (1999) claim that removing the second VP leads to an illusion of grammaticality.

Irrespective of the interpretation of these results (i.e., a grammaticality illusion in all ungrammatical conditions or only in V2), they are markedly different from those in Experiment 1. Consistent with the reading-time results from Frank et al. (2016), there appears to be a missing-VP effect for sentence judgments in English but not in Dutch. However, the difference between the outcomes of the two experiments could conceivably be due to differences between the two participant groups instead of the two languages. To directly test for an interaction between language and grammaticality in a within-subject design, Experiment 3 presents the Dutch and English items to native Dutch-speaking participants with high proficiency in English as a second language.

## Experiment 3: Dutch and English

### Method

#### Materials

Materials were the same as those of Experiments 1 and 2, except that the V1 and V3 conditions were not included to keep the total number of target items the same as in the first two experiments. Four lists were created such that each item occurred in both languages (Dutch and English) and in both forms (Grammatical: V0, and Ungrammatical: V2) across the lists but only once in each list. Languages were blocked such that each of these four lists first presented all Dutch items and then all English items. The first and last two sentences of a block were fillers, and two target items were always separated by at least one filler. A short text preceding each block stated the language of the upcoming sentences. This text was written in the language of that upcoming block. Four additional lists were constructed by reversing the presentation order of the four original lists, resulting in a total of eight lists (four starting with the Dutch block and four starting with the English block).

#### Procedure

Unlike Experiments 1 and 2, Experiment 3 was conducted in a controlled lab environment. Nevertheless, the rating task was administered using Qualtrics, as in the previous two experiments. All instructions were given in Dutch.

Following the rating task, participants filled out a short language background questionnaire, including self-ratings of English proficiency and amount of use on 7-point scales. As there was very little variability among participants, these ratings were not used for selecting participants or analyzing individual differences.

Next, participants performed two tests to ascertain their level of English proficiency. The first was the Vernon–Warden reading test (Hedderly, 1996), which consists of 42 sentence items with a missing word for which five options are provided. The task is to choose the correct word for as many items as possible within a 10-min time limit. The second was LexTALE (Lemhöfer & Broersma, 2012), a lexical decision task designed to measure English proficiency in non-natives. A complete experiment session took between 30 and 60 min.

#### Participants

Thirty-one native Dutch-speaking participants (23 females, age range 20–41 years, mean age 25.5) who self-identified as speakers of English as a second language were recruited via the Radboud University participant registration system. They received a 10 euro gift voucher for their participation. 23 participants reported knowledge of a third or fourth language, mostly Frisian and local dialects of The Netherlands. All participants completed the experiment and none indicated any awareness of the missing-VP effect or grammaticality illusions.

One participant with very low Vernon–Warden score was excluded from further analysis.^2^ All remaining participants scored in the top 10–50% for adult natives on the Vernon–Warden test, indicating that they have near-native English-reading skills. LexTALE scores ranged between 61.25 and 100% (mean: 83.5%), which classifies the participants as upper intermediate or advanced/proficient second language users (see Lemhöfer & Broersma, 2012, Table 9).

### Results

As can be seen in Table 6, Dutch grammatical sentences are rated as more comprehensible and more acceptable than the sentences with a missing second verb phrase. The same does not seem to hold in English, where the difference in ratings between the two sentence types is much smaller. This was confirmed by an ordinal mixed-effects regression analysis (Table 7) which revealed a significant interaction between Grammaticality and Language, such that the grammaticality effect is larger in Dutch than in English. In fact, there was no significant effect for the English items, as can be seen in Table 7 from the absence of a simple effect of Grammaticality in the English (reference level) condition. Note, however, that this non-significance should be seen in the light of the lower number of participants (i.e., lower statistical power) compared to the previous experiment. Power analyses using the R package simr (Green, MacLeod, & Alday, 2017) estimated that powers for detecting effects the same size as in Experiment 2 are 0.34 and 0.52 for acceptability and comprehensibility, respectively. 3 These low values mean we cannot interpret the non-significance as evidence against a grammaticality effect on the English items.

Post hoc analyses that included English L2 proficiency as measured either by LexTALE or by the Vernon-Warden test did not reveal any main effect or interaction involving proficiency.

**Table 6:** Mean and by-subject standard error of ratings in Experiment 3, on a scale from 1 (very incomprehensible/unacceptable) to 7 (very comprehensible/acceptable)

Rated aspect	Language	Grammaticality condition	
		V0	V2
		M (SE)	M (SE)
Comprehensibility	Dutch	4.18 (0.23)	3.33 (0.24)
	English	4.00 (0.25)	3.93 (0.21)
Acceptability	Dutch	3.22 (0.24)	2.32 (0.17)
	English	3.01 (0.22)	2.84 (0.17)

**Table 7:** Fixed-effect coefficients *b* (with Grammaticality V0 and Language English as reference levels) and their *z*-scores and *p*-values from regression analysis of ratings in Experiment 3

Factor	Rated aspect					
	Comprehensibility			Acceptability		
	*b*	*z*	*p*	*b*	*z*	*p*
Language Dutch	0.28	0.78	$> 0.4$	0.20	0.51	$> 0.6$
Grammaticality V2	− 0.06	− 0.21	$> 0.8$	− 0.18	− 0.51	$> 0.6$
Dutch $\times$ V2	− 1.22	− 3.11	0.002	− 1.09	− 2.72	0.007

### Discussion

Experiment 3 replicated Experiments 1 and 2 with a within-subjects design and in a controlled lab setting. Again, the results showed that grammatical Dutch double-embedded sentences are considered more comprehensible and acceptable than ungrammatical versions in which the second verb phrase has been removed, but that the same is not the case in English. In short, the missing-VP effect appears in English but not in Dutch.

A possible objection to this conclusion is that Experiment 3 contains a confound between language and nativeness because all participants had Dutch as their first language (L1) and English as a second language (L2). If double-embedded structures are particularly hard to process in L2, this could lead to lower ratings in the English V0 condition compared to English V2. However, there are several reason why non-nativeness is unlikely to have caused the missing-VP effect in Experiment 3. First, if the sentences were harder to comprehend in L2, we would expect lower ratings for grammatical English compared to grammatical Dutch sentences. However, the data in Table 6 tell a different story: The cross-linguistic difference in grammaticality effect is mostly due to relatively high ratings in the English V2 condition. That is, the participants do not find English grammatical sentences (much) less acceptable and comprehensible than the Dutch equivalents, but they rate ungrammatical sentences more positively in English than in Dutch.

Second, the grammaticality effect for English sentences was much larger for the native speakers in Experiment 2 than the non-natives in Experiment 3. Although we should refrain from directly comparing the results of these two experiments because of the difference in setting (i.e., online versus in the lab), a larger missing-VP effect for natives than for non-natives is inconsistent with non-nativeness being the cause of the effect.

Third, insofar as the missing-VP effect is caused by working memory limitations, it is unlikely that non-nativeness plays a role, considering the evidence that working memory capacity does not differ between a bilingual’s two languages (Keijzer, 2013; Lanfranchi & Swanson, 2005; Osaka & Osaka, 1992; Osaka, Osaka & Groner, 1993).

Finally, a more fundamental reason to disprefer the non-nativeness account of the missing-VP effect is that this account is not parsimonious as it requires an alternative explanation for the same effect in native English speakers.

In Experiment 2, grammatical (V0) English sentences received lower ratings than ungrammatical (V2) items; an effect we did not replicate in the English stimuli of Experiment 3. Assuming that this is not simply due to lower statistical power, an interesting possibility is that it is caused by language transfer; the phenomenon that native-language properties affect processing in a second language. Specifically, the native Dutch participants would apply their successful strategies for processing Dutch double-embedded structures when reading in English. However, a much simpler explanation is that these participants were more attentive to the task and tried harder to parse the sentences, either because they were not reading in their native language or because they were tested in a lab environment. Consistent with the latter explanation, Gibson and Thomas’s (1999) lab study also did not find any significant difference between conditions V0 and V2, using very similar materials.

## General discussion

Vasishth et al. (2010) and Frank et al. (2016) demonstrated that the missing-VP effect on reading times is language dependent, in that it appears in English but not in German or Dutch. The current results provide further support for this finding and expand on it in three respects. First, we showed that the interaction between grammaticality (second VP present or missing) and language (Dutch or English) is not limited to reading times but also appears as a subjective illusion in sentence ratings: Dutch sentences with a missing VP were judged to be significantly less comprehensible and acceptable than their grammatical counterparts, whereas the same was not the case (or even reversed) for English sentences.

Second, we found that the cross-linguistic difference is also present for sentences whose propositional content (the “who-does-what-to-whom”) is apparent from the semantic relations between agents, patients, and actions. In contrast, understanding the materials of Vasishth et al. (2010) and Frank et al. (2016) required a full syntactic analysis because the nouns and verbs used in these sentences made any agent–action–patient triplet semantically possible.

Third, we compared among three ungrammatical conditions, corresponding to each of the three VPs being removed. Such a comparison was not available to Vasishth et al. (2010) and Frank et al. (2016) because the absence of semantic constraints in their stimuli made it impossible to tell which of the three VPs was missing. Our results for English were consistent with Gibson and Thomas (1999) in that ratings were higher in the V2 condition than when one of the other two VPs was removed. There was no sign of such a difference for Dutch (see Table 2), further strengthening the conclusion that the missing-VP effect does not arise in that language.^4^

Unlike the previous missing-VP sentence rating studies (Christiansen & MacDonald, 2009; Gibson & Thomas, 1999; Gimenes et al., 2009; Häussler & Bader, 2015), we had participants rate both acceptability and comprehensibility of the stimuli. Results were nearly identical for these two measures, which suggests there may be only one underlying cognitive factor at work. Alternatively, the convergence could have been caused by a type of anchoring effect (Tversky & Kahneman, 1974) in which a participant’s rating on the first scale biases the response on the second.

### Explaining the missing-VP effect

Three explanations of the missing-VP effect have been proposed in the literature. First, the structural forgetting account (Gibson & Thomas, 1999) claims that encountering a double-embedded structure results in working memory overload which leads to one of VP predictions to be forgotten. According to Gibson’s (1998) Syntactic Prediction Locality Theory, most memory is freed up if it is the prediction of the second VP that is dropped, which explains why only condition V2 of Experiment 2 results in relatively high comprehensibility and acceptability ratings. As explained in the Introduction, speakers of a verb-final language are more accustomed to keeping verb predictions in working memory and, according to Vasishth et al. (2010), this explains why the missing-VP effect does not occur in such languages.

Second, the interference account (Häussler & Bader, 2015) claims that the missing-VP effect is not caused by forgetting but by memory interference. More specifically, upon encountering the second verb phrase, there are two possible attachment sites (the first and second noun phrase) and a grammaticality illusion can occur when the first noun phrase instead of the second is incorrectly retrieved from memory. The first VP does not lead to such confusion because, at that point, the final noun phrase, to which it is to be attached, is still active in working memory. According to Häussler and Bader (2015), the reason why no grammaticality illusion arises when the third VP is deleted is that the first items of a list are more strongly represented in working memory than later items (except for the most recent ones) — this is the primacy effect that is well known from the working-memory literature (e.g., Page & Norris, 1998). If the second VP is correctly attached to the second noun phrase, the absence of a third noun phrase is detected relatively easily because of the first noun phrase’s primacy advantage. The advantage of the interference account over Gibson and Thomas’s (1999) structural forgetting account is that the former follows naturally from general properties of working memory rather than from a particular idea about syntactic complexity, such as the Syntactic Prediction Locality Theory.^5^

Third, according to the language statistics account (Frank et al., 2016), the language’s statistical word-order patterns are central to the occurrence of the missing-VP effect. This can explain why English behaves differently from Dutch and German, irrespective of the participant’s native language. As explained in the Introduction, the occurrence of consecutive VPs are more probable in verb-final than verb-initial languages. Sensitivity to such statistics can cause the third VP to be highly unexpected in English, while it is much less surprising in Dutch/German. Computational simulations using neural networks and other statistical models have shown that this is indeed a viable explanation of the cross-linguistic difference (Engelmann & Vasishth, 2009; Frank et al., 2016; Futrell & Levy, 2017). When these models have learned the statistical word-order patterns from a large corpus of English texts, they estimate higher word probabilities (corresponding to faster reading; Hale, 2001; Levy, 2008) in double-embedded English sentences when one verb phrase is removed. When the models are trained on a Dutch or German corpus, however, they estimate higher word probabilities in the grammatical Dutch/German sentence condition. In short, the models predict that the missing-VP effect on reading times arises in English but not in Dutch or German. Whether they predict the same cross-linguistic difference for a sentence rating task depends on whether such ratings correlate with probabilities. Recent research suggests that they do (Lau et al., 2017) so our results are in line with the language statistics account.

None of the three accounts on its own can straightforwardly explain all the available empirical data. For structural forgetting to explain why native Dutch and German speakers display the illusion in English, it needs to assume that working memory capacity is language specific, something that is highly unlikely (see the “Discussion” of Experiment 3, and references therein). Likewise, the interference account is unable to explain why the same sentence structures yield opposite effects in verb-initial (English and French) versus verb-final (German and Dutch) languages. The language statistics account is hard pressed to explain why dropping the first or third VP does not result in a grammaticality illusion in English.

The simplest model that can explain the current results may be a hybrid statistical and working-memory account (see Christiansen & Chater, 2016, Ch. 7, for a similar view). Primacy and recency effects lead to stronger working memory activation of the first and last noun phrase, so the absence of their verb phrases is easily detected. For grammatical English double-embedded sentences, the low occurrence probability of three consecutive VPs leads to a sense of unacceptability (and slowdown in reading) on the third VP, even if it is syntactically required. In verb-final languages, in contrast, the third VP is less unexpected, and therefore, more acceptable. This acceptability, in turn, can lead to a (false) sense of comprehension, although it should be kept in mind that these very complex sentences are still not considered very acceptable or comprehensible, with average scores of just over 4 on a 7-point scale.

It may appear unrealistic to claim that syntactically correct (three-VP) English sentences receive a lower probability than ungrammatical two-VP structures, but the subjective (and implicit) probabilities assigned by the language comprehension system need not be based on complete and correct syntactic parses. As a case in point, Lau et al. (2017) found that the probabilities assigned by a context-free grammar display weaker correlation with acceptability ratings than probabilities from a recurrent neural network. The same has been reported for eye-tracking and EEG data (Frank & Bod, 2011; Frank, Otten, Galli, & Vigliocco, 2015).

### Ambiguity of Dutch relative clauses

As mentioned in the “Materials” section of Experiment 1, Dutch relative clauses are ambiguous between subject-relative (SR) and object-relative (OR) readings. That is, the Dutch sentence fragment “Het spannende boek dat de populaire schrijver publiceerde” (lit.: “The exciting book that the popular author published”) can in principle be interpreted to mean that the book published the author. This is because Dutch, unlike German, does not have case marking. We reduced the amount of ambiguity by having each sentence start with an inanimate noun, which leads to an initial preference for the (intended) OR reading (Mak et al., 2002). Furthermore, only the third noun was plural so that the innermost embedding is unambiguously OR as soon as the plural verb is encountered. Semantically, too, the two relative clauses are more likely to be interpreted as OR. Nevertheless, the syntactic ambiguity of the first relative clause remains, which could lead to increased cognitive (or working memory) load compared to the English sentences in which any ambiguity is already resolved at the first word following the relative pronoun. However, this is unlikely to have caused the cross-linguistic difference because, to the extent that the missing-VP effect is caused by cognitive overload, an increased difficulty due to the Dutch ambiguity should make the grammaticality illusion even stronger. In contrast, what we find is its absence.

In Frank et al.’s (2016) Dutch sentences, no useful semantic information was present and all nouns were animate. Consequently, these structures could not easily be interpreted as ORs. For this reason, an adjective was inserted after each relative pronoun, which syntactically disambiguates towards an SR reading. Frank et al. (2016) argue that this could not have caused the reversal of the missing-VP effect compared to English, and our current results indeed confirm that the illusion is also absent in Dutch double-embedded OR clauses.

## Conclusion

In a series of three sentence-rating experiments, we have shown that the classical missing-VP effect reported by Gibson and Thomas (1999) holds up in English but not in Dutch. English double-embedded object-relative clauses from which the second VP is removed are considered to be more (or at least no less) acceptable and comprehensible than their grammatical counterparts, but this grammaticality illusion does not occur in similarly structured Dutch sentences. This cross-linguistic difference complements the earlier effects on reading times found by Vasishth et al. (2010) and Frank et al. (2016). Taken together, the reading-time and rating results form converging evidence for how word-order differences between languages can have subtle and perhaps unexpected consequences for sentence comprehension.

## Data availability

All data collected and analysed during this study are included in supplementary information files of this published article.

### Electronic supplementary material

Below is the link to the electronic supplementary material.

Supplementary material 1 (csv 13 KB)

Supplementary material 2 (R 3 KB)

Supplementary material 3 (csv 29 KB)

Supplementary material 4 (R 3 KB)

Supplementary material 5 (csv 11 KB)

Supplementary material 6 (R 3 KB)

## Appendix A: Target sentences

The 12 Dutch and English target sentences are listed below. Brackets indicate the words that were removed in the V1, V2, and V3 conditions.

1. Het eeuwenoude manuscript dat de nieuwe papiervernietiger die de studenten [in de bibliotheek gebruikten]$_\text {V1}$ [zonder enige moeite versnipperde]$_\text {V2}$ [miste een pagina]$_\text {V3}$.
  The ancient manuscript that the new paper shredder that the students [in the library used]$_\text {V1}$ [effortlessly shredded]$_\text {V2}$ [was missing a page]$_\text {V3}$.
2. Het liedje dat de moeder die de kinderen [lieten schrikken in de woonkamer]$_\text {V1}$ [heel erg graag zong]$_\text {V2}$ [ging over een verloren liefde]$_\text {V3}$. 
  The song that the mother who the children [scared in the living room]$_\text {V1}$ [thoroughly enjoyed singing]$_\text {V2}$ [was about a lost love]$_\text {V3}$.
3. Het spel dat het bedrijf dat de ouders [voor veel geld inhuurden]$_\text {V1}$ [heel goed organiseerde]$_\text {V2}$ [duurde heel de middag]$_\text {V3}$.
  The game that the company that the parents [hired for a lot of money]$_\text {V1}$ [organized seamlessly]$_\text {V2}$ [lasted the whole afternoon]$_\text {V3}$.
4. De bal die de bekende voetballer die zijn medespelers [egoïstisch vonden]$_\text {V1}$ [helemaal over het veld pingelde]$_\text {V2}$ [was lek]$_\text {V3}$.
  The ball that the famous soccer player who his team mates [thought selfish]$_\text {V1}$ [dribbled across the entire field]$_\text {V2}$ [had a puncture]$_\text {V3}$.
5. Het vrijstaande huis dat makelaar die de enthousiaste geïnteresseerden [heel vaak belden]$_\text {V1}$ [het liefst zou verkopen]$_\text {V2}$ [stond te koop]$_\text {V3}$.
  The detached house that the real-estate agent who the enthusiastic house hunters [called a lot]$_\text {V1}$ [would rather sell]$_\text {V2}$ [was for sale]$_\text {V3}$.
6. Het artikel dat de redacteur die de journalisten [niet geschikt vonden voor zijn functie]$_\text {V1}$ [enthousiast corrigeerde]$_\text {V2}$ [bevatte een aantal spellingsfouten]$_\text {V3}$.
  The article that the editor who the journalists [thought was unsuitable for the function]$_\text {V1}$ [enthusiastically corrected]$_\text {V2}$ [contained a number of spelling mistakes]$_\text {V3}$.
7. Het spannende boek dat de populaire schrijver die de recensenten [nauwlettend bekritiseerden]$_\text {V1}$ [met veel vertrouwen publiceerde]$_\text {V2}$ [miste een aantal pagina’s]$_\text {V3}$.
  The exciting book that the popular author who the reviewers [meticulously criticized]$_\text {V1}$ [very confidently published]$_\text {V2}$ [was missing a number of pages]$_\text {V3}$.
8. Het ijzeren standbeeld dat de kunstenaar die de leerlingen [hielpen]$_\text {V1}$ [met veel liefde smeedde]$_\text {V2}$ [was vrijdagnacht plots gestolen]$_\text {V3}$.
  The iron sculpture that the artist who the pupils [helped]$_\text {V1}$ [lovingly forged]$_\text {V2}$ [was suddenly stolen on Friday night]$_\text {V3}$.
9. Het oude muziekstuk dat de dirigent die de muzikanten [al een tijd verafschuwden]$_\text {V1}$ [vol overgave dirigeerde]$_\text {V2}$ [bestond uit drie delen]$_\text {V3}$.
  The old music piece that the composer who the musicians [have abhorred for some time]$_\text {V1}$ [passionately conducted]$_\text {V2}$ [consisted of three parts]$_\text {V3}$.
10. De film die de strenge regisseur die de acteurs [goed gehoorzaamden]$_\text {V1}$ [levendig visualiseerde]$_\text {V2}$ [werd illegaal gedownload]$_\text {V3}$.
  The film that the strict director who the actors [obeyed well]$_\text {V1}$ [vividly visualized]$_\text {V2}$ [was downloaded illegally]$_\text {V3}$.
11. De luxe kamer die de schoonmaakster die de gasten [niet aardig vonden]$_\text {V1}$ [twee maal per dag dweilde]$_\text {V2}$ [werd niet zo vaak verhuurd]$_\text {V3}$.
  The luxury room that the cleaner who the guests [didnt like]$_\text {V1}$ [mopped twice a day]$_\text {V2}$ [was not rented out often]$_\text {V3}$.
12. Het chique restaurant dat de klant die de obers [uitstekend bedienden]$_\text {V1}$ [een goede beoordeling had gegeven]$_\text {V2}$ [won een prijs voor klantvriendelijkheid]$_\text {V3}$.
  The chic restaurant that the customer who the waiters [excellently served]$_\text {V1}$ [had given a great review]$_\text {V2}$ [won a prize for its hospitality]$_\text {V3}$.

## Appendix B: Additional analyses

Tables 8 and 9 present the regression results of Experiments 1 and 2, respectively, when data is excluded from participants who did not rate all 12 target items. Table 10 presents the regression results of Experiments 3 when all participants are included.

**Table 8:** Fixed-effect coefficients *b* (with condition V0 as reference level) and their *z* scores and *p* values from regression analysis of ratings in Experiment 1.

Condition	Rated aspect					
	Comprehensibility			Acceptability		
	*b*	*z*	*p*	*b*	*z*	*p*
V1	− 0.88	− 3.10	0.002	− 0.90	− 2.32	0.020
V2	− 1.03	− 3.26	0.001	− 0.85	− 2.15	0.031
V3	− 1.61	− 5.44	< 0.0001	− 1.57	− 4.64	< 0.0001

**Table 9:** Fixed-effect coefficients *b* (with condition V0 as reference level) and their *z*-scores and *p*-values from regression analysis of ratings in Experiment 2.

Condition	Rated aspect					
	Comprehensibility			Acceptability		
	*b*	*z*	*p*	*b*	*z*	*p*
V1	−0.15	−0.54	$> 0.5$	0.47	1.27	> 0.2
V2	0.68	2.26	0.024	0.64	1.75	0.079
V3	−0.42	−1.45	> 0.1	− 0.30	− 0.85	> 0.3

**Table 10:** Fixed-effect coefficients *b* (with grammaticality V0 and language English as reference levels) and their *z*-scores and *p*-values from regression analysis of ratings in Experiment 3.

Factor	Rated aspect					
	Comprehensibility			Acceptability		
	*b*	*z*	*p*	*b*	*z*	*p*
Language Dutch	0.28	0.76	> 0.4	0.24	0.61	> 0.5
Grammaticality V2	− 0.06	− 0.19	> 0.8	− 0.12	− 0.36	> 0.7
Dutch $\times$ V2	− 1.16	− 3.01	0.003	− 1.16	− 2.95	0.003

## Footnotes

1 Frazier (1987) found that 31% of such syntactically unambiguous Dutch object-relative clauses are nevertheless incorrectly interpreted as subject relatives. Unlike our stimuli, however, the items from Frazier’s study were not semantically biased towards an OR reading.

2 Nearly identical results were obtained when this participant was included; see Appendix B.

3 simr does not handle ordinal models so these results are based on linear mixed-effect analyses instead.

4 If the V2 condition had resulted in higher ratings than V1 and V3, this could have been interpreted as evidence for a missing-VP effect that occurs in only some of the participants.

5 Both accounts would predict that readers with higher working-memory capacity display a weaker missing-VP effect. For this reason, it is of interest to investigate if age (as a proxy of working-memory capacity) interacts with grammaticality. However, post hoc analyses including age as a predictor revealed no significant interactions in any of the experiments.
